# Choroidal and Cutaneous Metastasis from Urothelial Carcinoma of the Bladder after Radical Cystectomy: A Case Report and Literature Review

**DOI:** 10.1155/2014/491541

**Published:** 2014-11-06

**Authors:** Yozo Mitsui, Naoko Arichi, Keita Inoue, Miho Hiraki, Shigenobu Nakamura, Takeo Hiraoka, Noriyoshi Ishikawa, Riruke Maruyama, Hiroaki Yasumoto, Hiroaki Shiina

**Affiliations:** ^1^Department of Urology, Shimane University Faculty of Medicine, 89-1 Enya-cho, Izumo 693-8501, Japan; ^2^Department of Pathology, Organ Pathology Unit, Shimane University Faculty of Medicine, 89-1 Enya-cho, Izumo 693-8501, Japan

## Abstract

Bladder cancer is the second most common genitourinary malignancy and has variable metastatic potential; however, choroidal and cutaneous metastases are extremely rare. Generally, a patient with these uncommon metastases has a very poor prognosis. We present a bladder cancer patient with a visual disorder in the right eye and multiple nodules on head and lower abdomen that developed 17 months after a radical cystectomy. These symptoms were determined to be caused by choroidal and cutaneous metastasis of bladder cancer. Although two cycles of combination chemotherapy were performed, the patient died 5 months after diagnosis of multiple metastases.

## 1. Introduction

Bladder cancer is the second most common genitourinary malignancy with urothelial carcinoma and comprises 90% of all primary bladder cancers. Approximately 50% of affected patients will develop local recurrence and/or metastatic disease after undergoing radical cystectomy [1]. Bladder cancer has variable metastatic potential and the most common metastatic sites are the lymph nodes, liver, lungs, and bones [2]. However, orbital metastasis of bladder cancer is extremely rare with fewer than 25 cases reported, only 5 of which showed metastasis in the choroid [3–7]. In addition, cutaneous metastasis of bladder cancer is also quite uncommon [8]. Herein, we present a patient with bladder cancer who developed choroidal and cutaneous metastases 17 months after a radical cystectomy. We also discuss some previous reports presented in the literature.

## 2. Case Presentation

**Figure 1:**
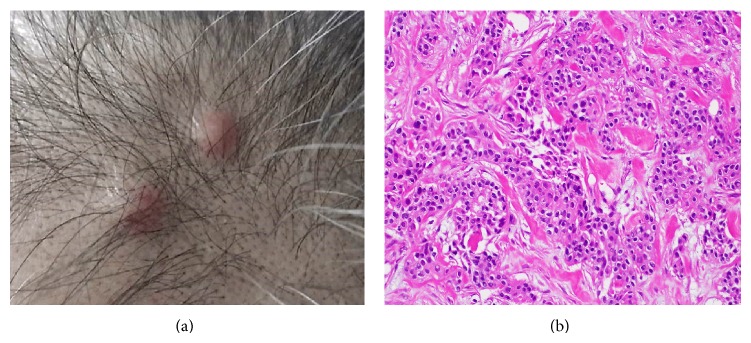
(a) Clinical image showing multiple 1 cm sized erythematic nodules on the head. (b) The excisional biopsied skin sample from the head showing individually scattered and nested pleomorphic tumor cells in the dermis, which is consistent with metastatic urothelial carcinoma (H&E staining ×200).

A 48-year-old male came to our hospital in September, 2011, because of a visual disorder in the right eye and general fatigue. He had undergone a radical cystectomy with an ileal neobladder construction and 2 courses of combination (gemcitabine/cisplatin: GC) chemotherapy for high-grade urothelial carcinoma of the urinary bladder (pT3aN0M0) 17 months previously. A physical examination revealed multiple erythematous nodules measuring 10 mm on the head and lower abdomen (Figure 1(a)). Excisional biopsy of the cutaneous nodule on the head revealed extensive infiltration of a high-grade urothelial carcinoma in the epithelium (Figure 1(b)).

**Figure 2:**
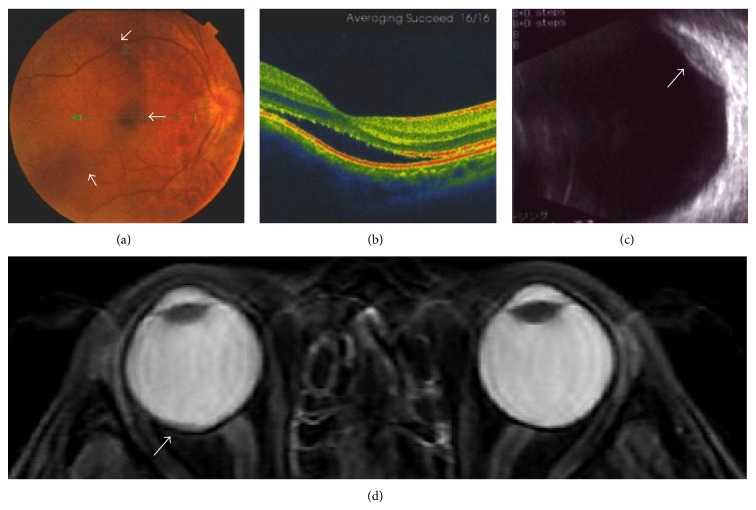
An elevated choroidal neoplasm. (a) Fundoscopic findings of the right eye demonstrating choroidal metastasis (arrows). (b) Optical coherence tomographic findings showing subretinal fluid in the inferior aspect of the lesion compromising the fovea. ((c) and (d)) Ultrasound and MRI T2-weighted images demonstrating an elevated choroidal mass (arrow).

Contrast-enhanced computed tomography scanning revealed multiple lung and bone metastases, in addition to cutaneous metastases. To evaluate the cause of the visual disorder, fundoscopic, optical coherence tomographic, ultrasound, and magnetic resonance imaging (MRI) examinations were performed. Fundus imaging of the right eye in an optical coherence tomographic examination showed a yellow choroidal lesion associated with subretinal fluid (Figures 2(a) and 2(b)), while ultrasound and MRI T2-weighted images of the right orbit showed an elevated choroidal mass (Figures 2(c) and 2(d)). On the basis of these findings and the clinical course, we diagnosed the choroidal mass as a metastatic tumor of the urinary bladder.

The patient was admitted to our hospital and 2 courses of combination chemotherapy (methotrexate at 50 mg/kg of body weight, vinblastine at 3 mg/kg of body weight, doxorubicin at 30 mg/kg of body weight, and carboplatin to reach an area under the curve value of 6) were performed, though it was not effective for any of the metastatic lesions. The patient died 5 months after diagnosis of multiple metastases of urothelial carcinoma of the urinary bladder due to the widespread metastasis. An autopsy could not be performed because his family refused it. Therefore, we could not pathologically diagnose the choroidal tumor as a metastatic tumor.

## 3. Discussion

Bladder cancer most commonly metastasizes to the regional lymph nodes, liver, lungs, and bones [2]. In contrast, choroidal or cutaneous metastasis is extremely rare. The occurrence of choroidal metastasis from all forms of carcinomas ranges from 2.3% to 9.2%, with the most common primary sites being the breasts and lungs [9]. However, since the first case reported in 1974 [4], there have been only 5 additional cases of metastasis to the choroid from urothelial carcinoma of the bladder prior to the present case [5–7]. In addition, the incidence of cutaneous metastases from bladder cancer was reported to be only 0.84% [8]. Therefore, this case is extremely rare, since the patient developed both metastases concurrently.

Melanoma and metastatic tumors are the most common types of malignant choroid tumors [10]. Due to difficulty with pathological diagnosis, these tumors are identified by history of present or prior malignancies and results of an ophthalmological examination with slit-lamp biomicroscopy and indirect ophthalmoscopy [9]. In addition, optical coherence tomographic and ocular ultrasound and MRI examinations may aid in the diagnosis of choroidal metastasis. In the present case, MRI T2-weighted imaging of the orbits demonstrated a well-circumscribed subretinal low intensity mass, which was consistent with a metastatic tumor [11]. Thus, a multidisciplinary approach with several imaging modalities seems to contribute to precise diagnosis of choroidal metastasis.

In cases of urological cancer with cutaneous metastasis, infiltrated plaque or nodule is the most common gross appearance [8]. However, these are not distinctive and may mimic many common dermatologic disorders [12, 13]. Skin biopsy findings can be useful for differential diagnosis because more than 90% of urothelial carcinoma cases with cutaneous metastasis have urothelial histopathology [14]. In the present case, we made a pathological diagnosis based on skin biopsy findings.

The presence of choroidal or cutaneous metastasis suggests a late manifestation of systemic spreading of cancer. Indeed, more than half of the affected patients already have systematic metastasis at the time of diagnosis of choroidal metastasis [15–17]; thus, the treatment of choice is systemic chemotherapy if the primary tumor is susceptible to anticancer agents. However, choroid or cutaneous cancer cell metastasis presents a poor prognosis [5–8]. Shimomura et al. [18] reported that treatment with epidermal growth factor receptor tyrosine kinase inhibitor (EGFR-TKi) is promising for ocular metastasis of non-small-cell lung cancer harboring an EGFR mutation. Although EGFR-TKi may be ineffective for bladder cancer, some of the major molecular targeting drugs currently available seem to be effective [19]. In the near future, these molecular target therapies may bring about improved outcomes for patients with choroidal or cutaneous metastasis.

New multitherapeutic approaches to bladder cancer, including several systemic combination chemotherapies, will provide new insight into improving the prognosis of bladder cancer patients [19]. On the other hand, Spector et al. [20] showed that uncommon bladder cancer metastasis may be a result of increased longevity in successfully treated patients. Our patient received 2 courses of GC chemotherapy following a radical cystectomy and developed choroidal and cutaneous metastases 17 months after treatment. We suggest that urologists must be aware of these rare metastases in patients with bladder cancer along with the development of new therapeutic approaches.
